# ESR Essentials: basic physics of MR safety—practice recommendations by the European Society for Magnetic Resonance in Medicine and Biology

**DOI:** 10.1007/s00330-024-10999-8

**Published:** 2024-08-13

**Authors:** Love Engström Nordin, Karin Åberg, Johan Kihlberg, Titti Owman, Boel Hansson, Isabella M. Björkman-Burtscher, Cecilia Petersen, Peter Lundberg

**Affiliations:** 1 Department of Neurobiology, Care Sciences and Society (NVS), Karolinska Institutet, and Department of Diagnostic Medical Physics, Karolinska University hospital, Solna, SE-171 76 Stockholm Sweden; 2 Center for Experimental and Biomedical Imaging in Örebro (CEBIO), Faculty of Medicine and Health, Örebro University, SE-701 82 Örebro, Sweden; 3 Department of Radiology and Medical Physics, Örebro University Hospital, Region Örebro County PO Box 1613, Örebro, SE-701 16 Sweden; 4 Department of Radiology in Linköping and Department of Health Medicine and Caring Sciences, Linköping University, Linköping, Sweden; 5 Center for Medical Image Science and Visualization (CMIV), Linköping University, Linköping, Sweden; 6 Department of Diagnostic Radiology, Clinical Sciences, Lund University, Lund, Sweden; 7 Department of Radiology, Clinical Sciences, Sahlgrenska Academy, University of Gothenburg, Gothenburg, Sweden; 8 Department of Radiology, Sahlgrenska University Hospital, Region Västra Götaland, Gothenburg, Sweden; 9 Department of Radiation Physics, and Department of Health, Medicine and Caring Sciences, Linköping University, Linköping, Sweden

**Keywords:** Magnetic resonance imaging, Static magnetic field, Radiofrequency field, Time-varying magnetic field gradients, Risk-benefit assessment

## Abstract

**Objectives:**

The use of magnetic resonance imaging (MRI) is safe from a long-term perspective since there are no known cumulative risks for patients or personnel. However, the technique comes with several acute risks associated with the powerful electromagnetic fields that are necessary to produce medical images. These risks include, among other things, a projectile hazard, loud noise, and the risk of heating. Safe use of MRI requires knowledge about the different hazards related to MRI and organizational structured work including the implementation of routines describing a safe workflow from the referral of a patient to the signed report. In this article, the risks associated with MRI are described along with suggestions for how each risk can be minimized or eliminated.

**Conclusion:**

The aim of this article is to provide support for the development of, and compliance with, MRI safety routines, and to work with the technique in a safe way. The scope of this treatise does not cover specific details of implant safety, however, the physical principles described can be applied to the risk assessment of implants.

**Key Points:**

- *Establish whether any MR contraindications apply to the patient.*
- *Evaluate means to deal with identified risks for both patients and personnel.*
- *It is imperative to always perform and document a risk-benefit assessment*.

## Key recommendations

- Establish whether any MR contraindications apply to the patient.
  *Are there any structured or unstructured electronic medical records or information that the patient has any implants, devices, or foreign objects in their body? If so, this information must without any exceptions, be known by the clinic that will perform the MR examination*.
- Evaluate means to deal with identified risks for both patients and personnel.
  *MR conditions for implants, devices, and foreign objects must be established; coping strategies for other contraindications must be established*.
- Perform and document a risk-benefit assessment.
  *Consider the identified risks associated with the intended examination, established coping strategies, and expected benefits in terms of diagnostic value and potential to reach or alter treatment strategies for the individual patient*.

The key recommendations are based on literature reviews of the fundamental physics of MR safety and expert opinion, as well as conditions provided by manufacturers.

Level of evidence = 5 (*Oxford Centre for Evidence-Based Medicine*).

## Introduction

The use of magnetic resonance imaging (MRI) in modern healthcare is both essential and increasing and often considered as a safe imaging procedure due to the lack of ionizing radiation. However, although there are no known cumulative risks associated with MRI there are several acute risks. To address the safety of patients, personnel, and equipment, an organizational structure including the implementation of strict routines and procedures by everyone involved in the process is required. Here we will focus on the essentials of MRI safety, primarily focusing on the risks related to the three electromagnetic fields in the MRI environment (Zones III and IV), methods for minimizing those risks, and related risk-benefit assessments.

## Introduction to the MR environment, particularly inside the bore of the scanner

The MR environment consists of an extremely strong static magnetic field (*B*_0_) that is always present. In most clinical MR scanners, the static magnetic field strength is either 1.5 T or 3 T, but both higher and lower field strengths exist. Due to the strong static magnetic field, the scanner room (also known as the examination room or magnet room) needs to be a strictly controlled area and only authorized persons should have access. Authorized persons are those who have undergone specific MR safety training. During scanning, two complementary electromagnetic fields are utilized, the time-varying gradient field (dB/dt) and a radiofrequency field (*B*_1_ or RF), each associated with different hazards. These two latter fields are primarily of concern for the patient since they do not reach very far outside of the scanner bore, but nonetheless, safety measures need to be taken.

The length of the electromagnetic coil that generates the strong static magnetic field in the MRI scanner approaches the length of the magnet housing. The gradient coils for generating the time-varying dB/dt in three orthogonal directions (*x*, *y*, and *z*) are somewhat shorter. Finally, the innermost coil in the “magnet” is the built-in RF coil (also known as the “body coil” or the “quadrature body coil”, the “QBC”). The length of the *B*_1_-coil is typically 50–70 cm in conventional clinical scanners. In the following sections, we discuss each of these electromagnetic fields separately, with a specific perspective on the safety aspects.

### Properties of and issues with *B*_0_

The static magnetic field, which is always present, includes three important characteristics:

1. *B*_0_, represents the maximum strength of the magnetic field achieved in the central volume of the magnet bore. The strong and homogenous magnetic field is often used to describe the scanner (typically 1.5 T or 3 T in conventional clinical scanners). The SI unit for describing the *B*_0_ magnetic field strength is T (for tesla, named after Nicola Tesla), and the maximum *B*_0_ allowed in clinical MR scanners is at present according to IEC 8 T [1].
2. The stray magnetic field (sometimes “fringe field” is used), i.e., the static field strength at different positions and distances from the center of the magnet (known as the isocenter). The stray field plot, provided by the manufacturer, shows the field strength at a specific distance from the isocenter.
3. The spatial field gradient (known as dB/dz, or SFG, describes the change in *static* magnetic field strength as a function of distance from the magnet) is sometimes confused with the time-varying gradient field, but the latter is something quite different. The spatial field gradient does not change with time, only the location in the vicinity of the magnet, and it indicates how the static magnetic field varies with distance at a specific location in the scanner room. The spatial gradient field is crucial for the attractive force that occurs with ferromagnetic objects. The SFG plot can be found in the scanner instructions for use (IFU). Ferromagnetic objects strive to move toward an increased density of magnetic field lines, or simply, closer to the magnet. Modern MR scanners with strong magnetic fields are actively shielded, meaning that the magnetic field decreases rapidly with increasing distance. The attractive forces are strongest near the entrances (both the front and rear) to the bore of the magnet, and they decrease gradually when moving towards the isocenter inside the bore where it reaches zero in the volume with a homogenous magnetic field.

**Fig. 1:**
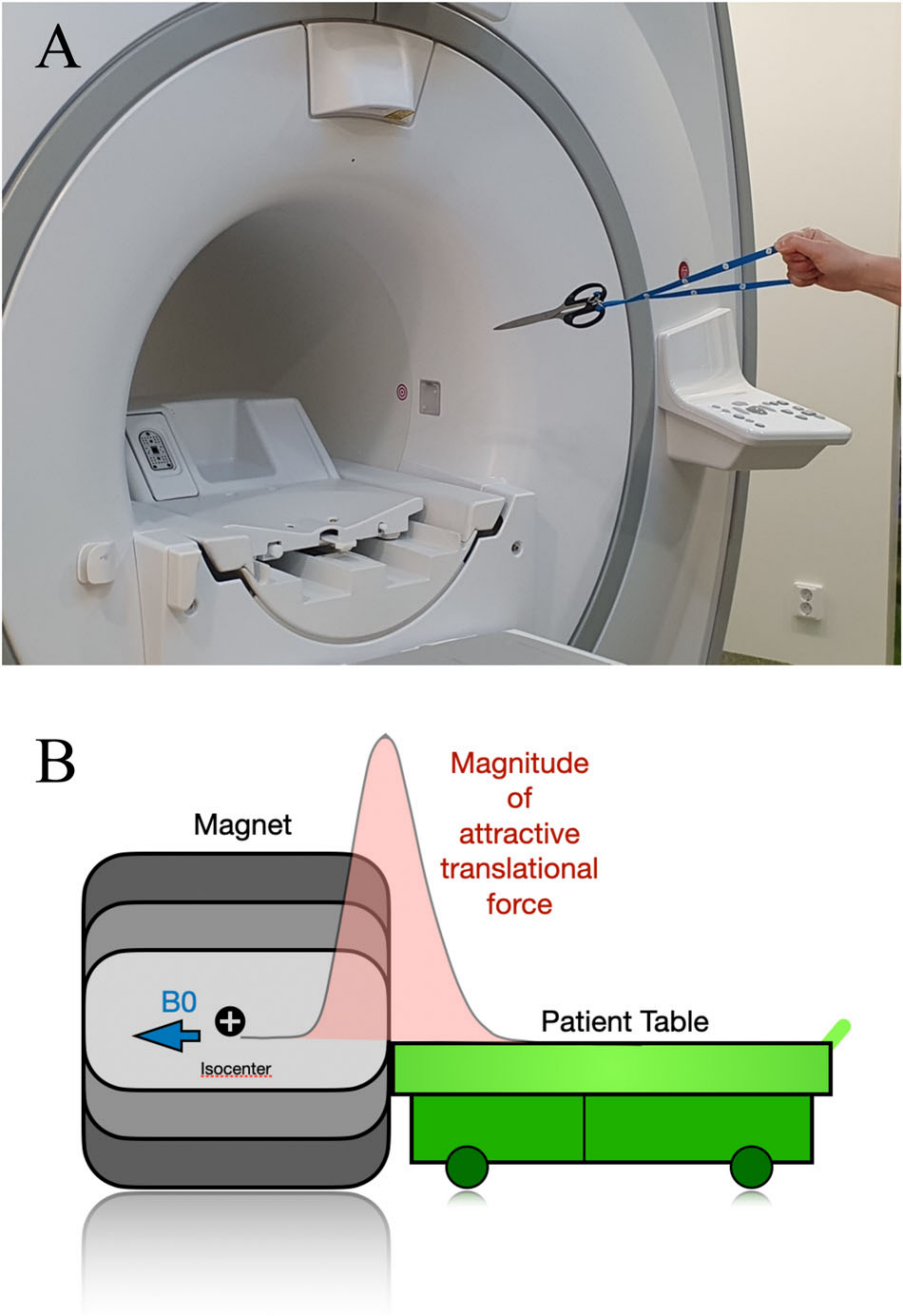
**A** A magnetic object dangerously close to a 3-T MR-scanner with a 70 cm diameter bore, illustrating both the translational (“pull”) and torque (“rotation” or aligning force) effects acting on the object. **B** A schematic representation of the magnitude of the attractive translational force (“pull”), which is maximal near the bore opening of the magnet. The illustration is based on Starck et al [14]. In contrast, the torque is typically at maximum within the bore

Ferromagnetic items will thus be attracted, accelerated, and act as projectiles if they approach the scanner (see Fig. 1). Besides that, elongated items will also rotate to align themselves in parallel with the magnetic field lines (related to a property called torque, or rotational force), which are oriented along the direction of the bore. For example, if a pair of ferromagnetic scissors accidentally is brought into the scanner room and sufficiently close to the magnet, the torque will make the scissors point toward the scanner and it will also become a projectile, with potentially fatal consequences for any person in the trajectory of this “projectile”.

If the ferromagnetic item is inside the body of a patient or personnel (for example an implant or shrapnel) the forces (translational as well as the rotational torque) will act on it and the consequences depend on where it is located and how it is attached. For example, is the item close to sensitive tissues such as nerve bundles or blood vessels? is it in soft tissue or is it attached to the bone?

It is of utmost importance to always strictly follow safety protocols to prevent MR-related incidents. All equipment that may need to be brought into the MRI-scanner room must be examined and labeled according to the applicable policy (with stickers stating that they are either MR safe or MR conditional). During the installation of a new MR scanner, consideration should be given to the stray field in all directions, as well as on the floors above and below.

Rapid motion in the static magnetic field may create sensory effects, for example, dizziness. Inside the scanner bore some individuals may experience “metallic taste” [2]. The static magnetic field may also cause vertigo and nystagmus (involuntary eye movement), both probably due to Lorentz force affecting the vestibular system [3]. These effects are both unusual and completely harmless and can be avoided by increasing the distance to the scanner, moving slower, keeping the eyes open, and focusing on something when positioned in the scanner bore [4].

The physical interaction between *B*_0_ and metal, as well as tissue, is the same for low-field MRI (below 1.5 T) and ultra-high-field MRI (above 3 T). However, most physiological effects will not be perceived at low fields. The attractive force and the torque at ultra-high field will most likely be stronger compared to common clinical systems [5, 6]. The attractive force is proportional to the product between spatial field gradients (dB/dz, dB/dy, and dB/dx) and the static magnetic field strength (*B*_0_) (until magnetic saturation). Torque towards *z* is proportional to the static magnetic field squared (${B}_{0}^{2}$) (Table 1). To assess the potential risks, the SFG-plot and stray magnetic field always need to be studied for each specific MRI scanner.

**Table 1:** The risks associated with each type of electromagnetic field in an MRI scanner are summarized along with suggested countermeasures to avoid accidents

Electromagnetic field	Risk	Countermeasure
Static magnetic field, *B*_0_	Attractive force $\propto {B}_{0}\frac{{dB}}{{dz}}$ (*B*_sat_ instead of B_0_ after magnetic saturation)	Avoid ferromagnetic metals
		Know $\frac{{dB}}{{dz}}$ of your scanner in relation to the position of any ferromagnetic object
		Switch to scanner with lower ${B}_{0}$
	Torque towards z $\propto {B}_{0}^{2}$ dependent on both shape and orientation	Check the shape of any ferromagnetic object. Torque = 0 for spherical objects, and it increases for elongated objects. Torque = 0 when parallel to *B*_0_, Torque = maximal at 45° to *B*_0_
		Switch to scanner with lower ${B}_{0}$
Time-varying gradient field, dB/dt	Loud noise	Hearing protection (e.g., > 28 dBA reduction)
	PNS	Scanning mode (normal/first level controlled)
	Induced voltage/current in active implants	Check implant slew rate limit
	Gradient induced vibrations	Not hazardous. Make sure to differentiate from RF-heating
Radiofrequency field, *B*_1_	Tissue heating	Scanning mode (limit SAR-exposure)
	Implant heating	Limit *B*_1+rms_
		Increase distance between implant-RF transmit coil
		Avoid resonance length (extra caution when > 10 cm) of conducting implant
	Tissue loop	Use insulating material/padding (≥ 1 cm)
	Proximity burns	Follow scanner IFU, keep a safe distance to scanner bore using padding (≥ 1 cm)
	Burn from equipment	Use MR-conditional equipment according to conditions. Avoid all kinds of loops, isolate cables from skin

*SAR* specific absorption rate (W/kg), *IFU* instructions for use, *PNS* peripheral nerve stimulation

## Properties of and issues with RF (*B*_1_)

During scanning an RF electromagnetic field (*B*_1_) is used. The RF field transfers energy to the body and it will therefore always cause heating to a small or large degree. Transmitted power is often expressed in SAR (*Specific Absorption Rate*) with the unit W/kg. SAR reflects the potential for heating of the patient and there are two important, clinically accessible operating SAR modes that needs to be considered (2 W/kg and 4 W/kg, respectively, for whole body SAR). The lower SAR level is called *Normal Operating Mode* as it is normally the default mode, whereas the higher SAR level is called *First Level Controlled Operating Mode*, which should only be used when required for certain image acquisitions.

Another more recently defined measure of the exposure  of the RF field is *B*_1+rms_. *B*_1+rms_ is in many ways a better and more accurate measure than SAR for assessing the risk for heating of implants. Many modern implants, especially active implants, are thus provided with MR conditions in terms of *B*_1+rms_, although many implanted devices are placed in the patient for life and maybe forgotten or undocumented, and the MR conditions of the devices may therefore not always be available from the manufacturer of the device. Such lack of information leads to an increased risk.

So which situations are associated with a high risk for tissue heating, either internally or externally? The RF field leads to the induction of currents that can cause burns when an electrically conductive loop is formed (for example, finger to the thigh or between the calves), and the burn may then appear in positions where the electrical resistance is the largest. The risk for burns can be minimized by ensuring that no skin surfaces touch each other with the help of at least 1 cm of insulating materials such as foam. Electrical currents can also be induced in conductive matter, when it is in close contact with the patient, e.g., sweat, damp clothing, or cables pose an additional risk of tissue burns. Another situation that might cause a burn is when an implant is of a certain length that resonates with the RF field, especially when it forms a narrow electrically conductive lead, with a projection on the *z*-axis (along the bore of the magnet) [7]. The  actual resonance length (sometimes called “antenna length”) of a conducting object depends on the dielectric properties of the surrounding tissue and the proton resonance frequency of the MRI scanner. It is therefore not straightforward to recommend specific safe or hazardous lengths, but extra caution should be applied to conducting objects that exceed 10 cm in particular in the *z*-direction, up to 3 T. Moreover, one must be especially cautious with leads that are abandoned, if they have been cut off to a length that deviates from the original length, and also if the lead has been capped at one end [8].

When there is a risk associated with the examination, a risk-benefit assessment needs to be carried out and measures taken to minimize the risk (such as limiting the RF field). Conductive particles in tattoo ink (such as iron oxide or carbon), or electrode gels can cause SAR hotspots that generate sufficient heat leading to cutaneous burns.

Another risk is the possibility of burns in proximity to the magnet bore, so-called ‘proximity burns’, most often pronounced at the end of the QBC [9]. Proximity burns occur because of near-field effects and are more probable at the point of maximum RF (the reactive near field, which is located very close to the transmitting coil surface) when the patient may be touching the scanner bore wall. This risk increases further when an electrically conducting material is close to the bore wall, acting as an antenna. To prevent burns, it is therefore important to ensure a distance between the patient and the bore of the scanner, and the manufacturers generally advise padding between the patient and the bore wall. The required distance to the bore wall is stated in the scanner IFU provided by the manufacturer; it is typically around 1 cm. Thus, it is important to study the safety recommendations provided by the manufacturer.

The risk of RF-induced heating effect depends, among other things, on the length of a conductor and the RF frequency of the MRI scanner. The RF frequency, which depends on *B*_0_, needs to be considered during risk assessments related to RF heating [5, 6]. Generally, the antenna length of any conducting material will decrease at ultra-high field MRI because of the higher frequencies, and correspondingly increase at low field MRI because of the lower frequencies.

## Properties of and issues with time-varying gradient field

In addition to the “static magnetic field gradients” (SFG), the patient is also subjected to exposure to time-varying gradient fields (dB/dt). The construction of an MR magnet is, in principle, like a loudspeaker. That is a varying input signal generates sound, or in the context of MRI, undesired noise. To prevent hearing damage due to high noise levels during MR examinations, everyone in the scanner room must wear appropriate hearing protection during image acquisition. Newer MRI scanners may offer noise reduction methods, but most manufacturers recommend dual hearing protection, i.e., a combination of earplugs and earmuffs.

Another potential problem of the time-varying field gradients is that they can lead to peripheral nerve stimulation (PNS), which are involuntary tingling sensation in certain muscles. Nerve stimulation can be experienced as unpleasant, but it is not dangerous in conventional clinical scanners. There are two clinically accessible operating modes also for dB/dt, *Normal Operating Mode* and *First Level Controlled Operating mode*, where the risk for PNS has been used to define the limits.

There are two additional risks associated with dB/dt in relation to implants. (i) Gradient-caused vibrations in implants can in some situations be confused by the patient as a feeling of warmth synchronized with scanning. (ii) Induction of electric currents caused by dB/dt can be harmful to patients/people with implants, for example, some types of neurostimulators (pacemakers, deep brain stimulators, etc.).

## Common causes of MR-accidents

**Fig. 2:**
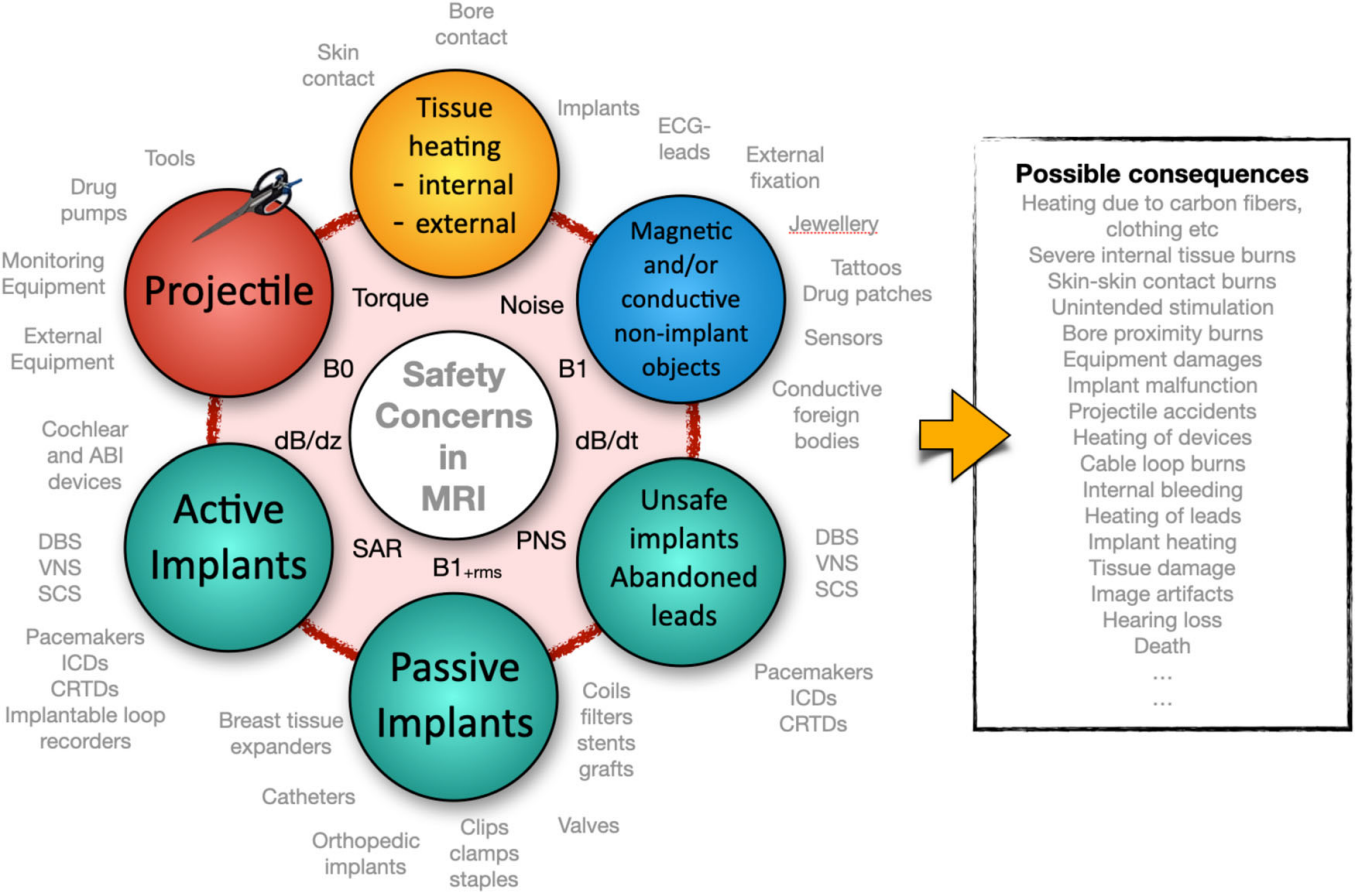
Safety concerns in magnetic resonance. Magnetic resonance is a very safe imaging modality, provided that the institution has implemented proper safety policies, routines, training, and site planning. (LEFT) The most important physical principles and related parameters affecting MR safety are shown in the red circle. The colored beads show different types of objects and processes that need to be considered prior to an examination. Note that all electrically conductive objects may be unsafe in the context of MR examinations, thus *not* only ferromagnetic metals. Examples of different types of objects that may interfere with a safe patient examination are shown in gray outside the circle of beads. (RIGHT) An overview of a number of possible consequences of improper application of MRI, originating from misunderstanding, or ignoring, the basic physical principles of this widely used imaging modality. ABI, auditory brainstem implant; DBS, deep brain stimulation; VNS, vagus nerve stimulation; SCS, spinal cord stimulation; ICD, implantable cardioverter defibrillator; CRTD, cardiac resynchronization therapy defibrillator; ECG, electrocardiogram; PNS, peripheral nerve stimulation; SAR, specific absorption rate; dB/dz, represents the spatial (static) field gradients in *x*, *y,* and *z* directions

**Fig. 3:**
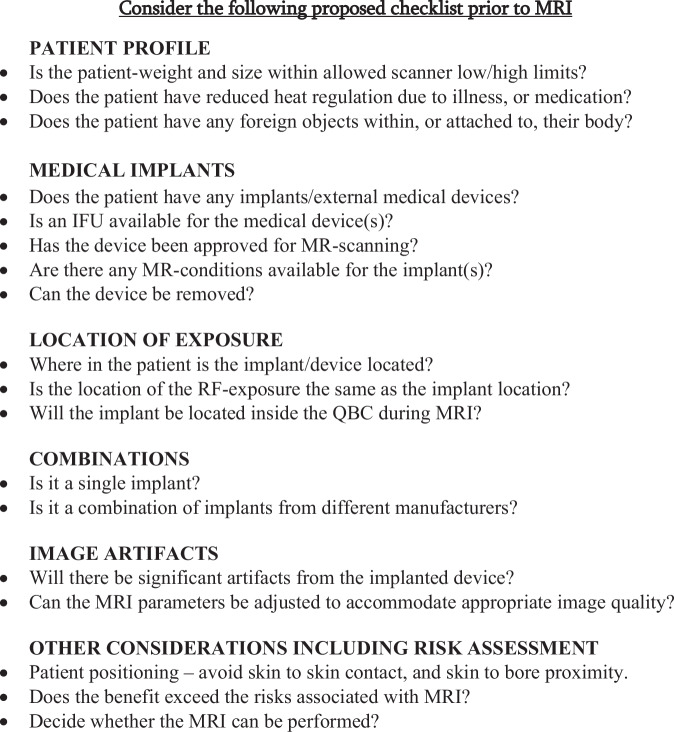
Abbreviated checklist. There are a number of potential risks associated with MR examinations, that need to be considered prior to performing an examination. The checklist here deals with a selection of those risks

Most of the reported incidents in Sweden involve projectile accidents, and the most often reported MR incidents in the USA and UK are tissue burns. However, and this is important to realize, there appears to be a very large degree of under-reporting [10]. The reasons for this are unclear as in most parts of the world it is a legal requirement to report all incidents, regardless of severity. See Figs. 2 and 3 for an overview of concerns regarding the consequences of improper use of MRI.

## Summary statement

Magnetic Resonance is entirely based on the use of non-ionizing electromagnetic fields and is therefore, in principle, completely safe. However, these fields are nevertheless very powerful and impose a number of conditions and circumstances that need to be considered with respect to patient safety. For example, does the patient have any implants and/or foreign objects, and if so, can they be safely scanned? Important considerations are therefore to accurately identify any implants, to follow the MR conditions stated by the manufacturer of the medical device, and to follow the recommended operation of the scanner as documented by the MR manufacturer. Besides the safety of the patients, one must not forget the safety of the personnel as well as the care for the equipment. Personnel need to be aware of, and adhere to, all the safety routines for their own, their colleagues’, and patients’ safety [11–13].

This article outlines some of the most important safety aspects that are associated with physics. Moreover, any incidents or accidents that unfortunately may occur, need to be reported without delay, and they must subsequently be analyzed in detail. The knowledge gained must then be transformed into improved safety practices. As medical devices are constantly being developed, and MR scanners are made stronger, more powerful, and more rapid, all personnel that work near or with MRI need to continuously update their knowledge on MR safety.

## Patient summary

There is a present and universal lack of structured and complete documentation of any implants that an individual patient may have. The patients should for that reason maintain their own life-long records of any surgical procedures performed and possible implants that they may have, as well as the existence of any other foreign bodies, whether ferromagnetic or not. Moreover, the patient must never on purpose conceal any such knowledge from the referring physician. A detailed risk-benefit analysis must always be performed prior to an MRI examination. The risks can be judged based on the underlying physics described here.

## Abbreviations

IFU: Instructions for use
QBC: Quadrature body coil
RF: Radiofrequency
SFG: Static magnetic field gradients
